# Stepwise slime mould growth as a template for urban design

**DOI:** 10.1038/s41598-022-05439-w

**Published:** 2022-01-25

**Authors:** Raphael Kay, Anthony Mattacchione, Charlie Katrycz, Benjamin D. Hatton

**Affiliations:** 1 Department of Mechanical and Industrial Engineering, University of Toronto, Toronto, Canada; 2 Department of Materials Science and Engineering, University of Toronto, Toronto, Canada; 3 John H. Daniels Faculty of Architecture, Landscape, and Design, University of Toronto, Toronto, Canada

**Keywords:** Computational models, Network topology, Microbiology, Structural biology, Systems biology

## Abstract

The true slime mould, *Physarum polycephalum,* develops as a vascular network of protoplasm, connecting node-like sources of food in an effort to solve multi-objective transport problems. The organism first establishes a dense and continuous mesh, reinforcing optimal pathways over time through constructive feedbacks of protoplasmic streaming. Resolved vascular morphologies are the result of an evolutionarily-refined mechanism of computation, which can serve as a versatile biological model for network design at the urban scale. Existing digital *Physarum* models typically use positive reinforcement mechanisms to capture meshing and refinement behaviours simultaneously. While these automations generate accurate descriptions of sensory and constructive feedback, they limit stepwise design control, reducing flexibility and applicability. A model that decouples the two “phases” of *Physarum* behaviour would enable multistage control over network growth. Here we introduce such a system, first by producing a site-responsive mesh from a population of nutrient-attracted agents, and then by independently calculating from it a flexible, proximity-defined shortest-walk to produce a final network. We develop and map networks within existing urban environments that perform similarly to those biologically grown, establishing a versatile tool for bio-inspired urban network design.

## Introduction

**Figure 1:**
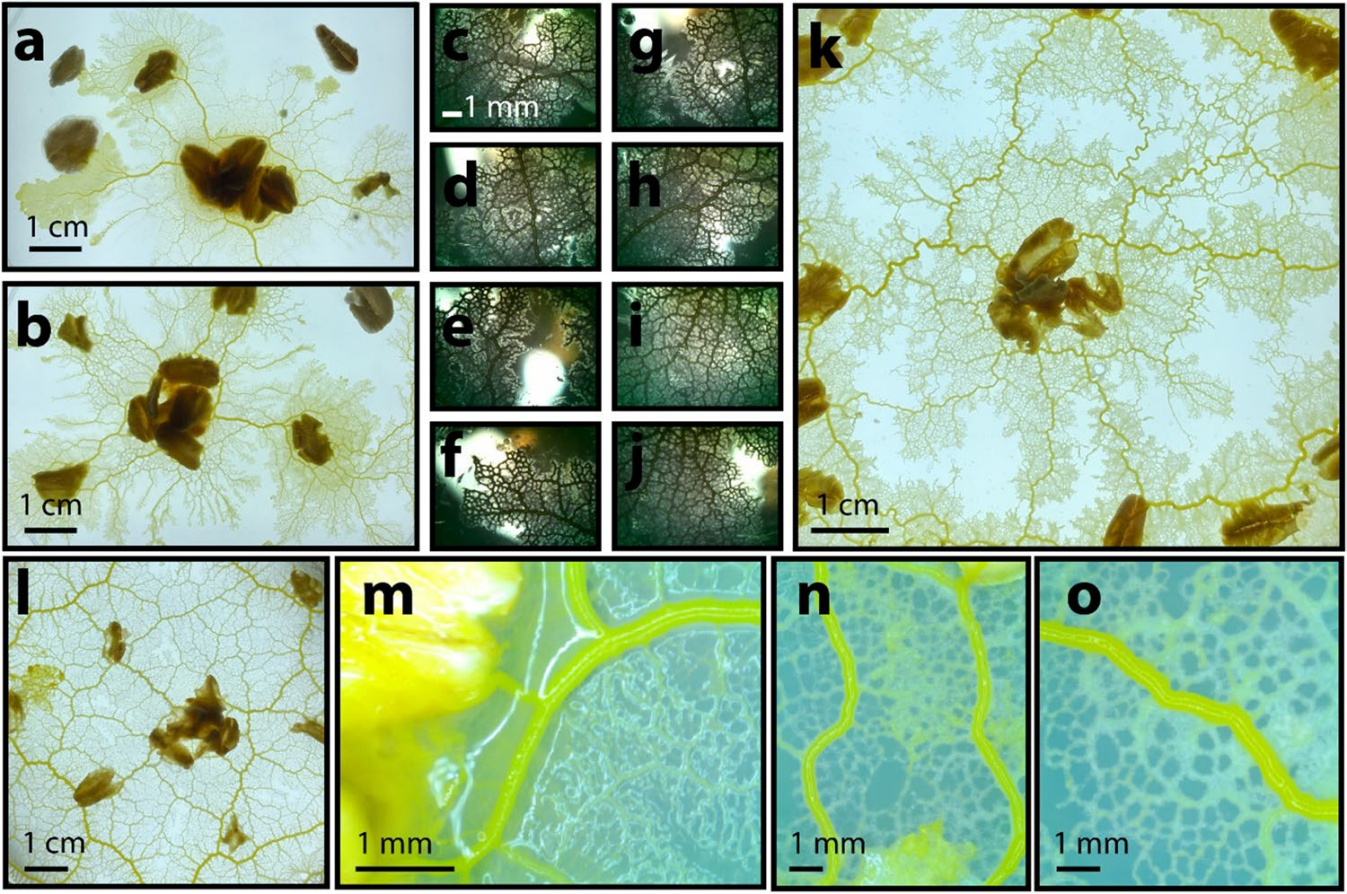
Intelligent growth network formation by the active plasmodial *Physarum polycephalum*. (**a**–**l**) Digital photographs and (**m**–**o**) optical microscope images of various stages of slime mould growth using oat flakes as nutrient sources. All images (other than **l**) photographed after 4 days of growth. (**l**) Photographed after seven days of growth.

**Figure 2:**
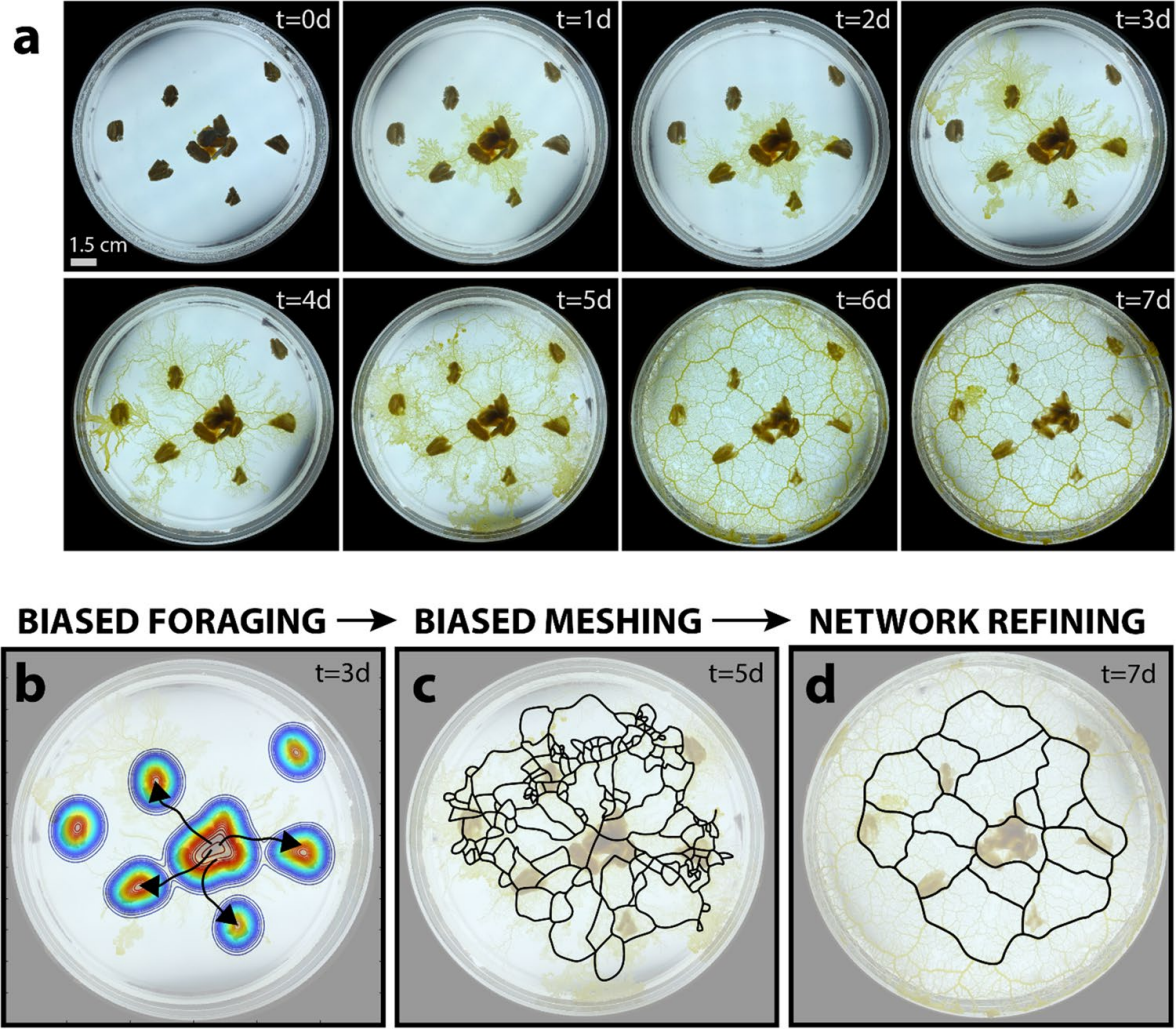
Simplified principles of plasmodial *Physarum polycephalum* growth. (**a**) Growth of the plasmodium across seven-day observation period. (**b**) Illustrated depiction of plasmodial growth according to food source proximity. This behaviour leads to mesh clustering (phase one). (**c**) Illustrated depiction of mesh clustering, with mesh regions illustrated. (**d**) Illustrated depiction of network refinement and shortest-path optimization (phase two), where only a refined network remains and is illustrated. Illustrations overlaid atop true photographs.

The true slime mould, *Physarum polycephalum*, is a single-celled amoeboid organism with a remarkable aptitude for design (Fig. 1). The slime mould cell grows (1 mm/h to 1 cm/h) as a tubular network of protoplasm, guided by the nutrient-rich environment within which it forages (Fig. 2a, t = 5 days)^1^. As *Physarum* encounters discrete food sources along its path, it develops from a preliminary mesh into a refined network, comprising both direct and indirect pathways between sources of nutrition (Fig. 2a, t = 7 days, Supplementary Fig. S1)^2^. Such a network is a remarkable accomplishment: while many biological organisms have established sophisticated neural circuitry to process, learn from, and adapt to sensory information, the slime mould has instead relied on a decentralized and fluidic form of adaptive physical computing^3,4^. The tubular and gel-like cytoskeleton of the organism acts as a spatially-varied molecular motor^5^, comprising many distinct oscillator units distributed across the cell. Each unit generates a wave-like contraction at a frequency dependent on both the neighbouring contraction frequency and the presence of attractants (e.g., food) and repellants (e.g., light) within its immediate environment^5–11^. Attractants (e.g., food) cause molecular receptor binding at the surface membrane of the organism^6^. This leads to an increase in the contraction frequency of the units nearest to the attractant, shuttling cytoplasmic fluids towards nutrient-rich regions^12^. Molecular binding also causes a local decrease in surface tension at the point of attraction, inducing a hydrostatic pressure difference, forcing cytoplasmic fluid to additionally flow towards nutrition^6^. Cell morphology is dictated by feedback loops of collective oscillation and cytoplasmic fluid flows, whereby the organism can simultaneously refine efficient links and starve and destroy redundancies^13^.

This reactive routing mechanism enables highly sophisticated behaviour: *Physarum* is able to navigate mazes with a minimum path length^14–16^, form proportional contacts between unique food sources for an optimal diet^17^, grow as a mathematical minimum tree^18^, remember where it has been^7^, anticipate regular events^19^, and even mimic with impressive accuracy the Tokyo rail system^20^ and Autobahn^21^. This non-neural, fluidic, biological computer—able to process new information, make informed decisions, and learn without the aid of a neural circuit—represents an intriguing computational model for unconventional data processing, problem solving, and memory storage^22^. Efforts to study and capture this capacity have been established, whereby *Physarum* gates have been leveraged to perform logical operations^23^, while *Physarum* cells have been explored as tactile, chemical, and colour sensors^24–26^, as well as processing chips^27^.

## *Physarum* for urban design

Slime mould can also crucially serve as a biological model for adaptive computational network design^20^. *Physarum*, like colonies of microorganisms^28,29^, fungal networks^30^, and wood-ant trails^31^, represents a highly-efficient and decentralized growth network. The biological principles that underpin the organization of this system have undergone thousands of cycles of evolution, and can provide fundamental insights for dealing with complex networking problems of our own (e.g., road planning^21,32^, railway planning^20^, wireless sensor routing^33^), and particularly at the scale of the city. Urban design—simplified here as the formal conceptualization of urban networks (e.g., roadways and railways)—has historically occurred without well-understood or well-defined criteria^20,34^. Major arterial Paris roadways, for instance, were famously introduced in the mid-1800s on the basis of modernization and circulation^35,36^. While grid-like urban morphologies were developed in Barcelona, driven in large part by political forces like land-ownership^37^. Ebenezer Howard’s Garden City was conceptualized as a utopian revolution against industrialized urban form^38^. Yet, around the same time, Le Corbusier conceptualized the motorway networks of Pessac, France to oppositely prioritize automobile and ‘industrial’ efficiency^39^, which is a trend that has continued in the planning of many modern city networks in the United States^40^.

In the 1960s, architects at the Institute for Lightweight Structures in Stuttgart began experimenting with self-assembling natural materials, hoping to find generalizable physically-optimized solutions for urban network design. Frei Otto, who pioneered theories of material computation in design, used soap films to connect a set of points as a minimal surface, generating what can be extrapolated as a minimal-spanning network (or Steiner Tree) for given urban geometries^41,42^. This work has inspired a series of computational explorations since^41–44^, focusing primarily on digital computation of urban networks.

*Physarum*, as a biological computer, has also been tasked with similar urban design problems. Slime moulds have ‘redesigned’ Iberian roadways^45^, establishing transport networks that differ from existing road segments, but that maintain comparable transport performance. Similarly, *Physarum* has ‘rerouted’ the M6 motorway through Newcastle^32^, and provoked questions regarding the redundancy of the Mexican highway system^46^. Indeed, while the organism can impressively reconstruct existing anthropically-designed urban networks^20^, the very discrepancies between city infrastructures, and their biologically-grown *Physarum* analogues, have proven equally crucial for understanding characteristics of both urban network planning and *Physarum* intelligence^20,21,32,46^.

## Physarum modelling

Models, developed in order to both understand and mimic the organism’s behaviour, have emphasized aspects of attractor-based foraging^47^, environment-driven morphology^1^, and flux-induced cytoplasmic streaming (i.e., flow)^20,48,49^. For example, Wu et al. developed networks by modelling cellular foraging along the gradient of nutrient attractors and anti-attractors^47^. Spanning trees grew serially, as a growth point moved along a nutrient-poor terrain under the influence of a field source. In another model, two agent-like *Physarum* populations searched across a nutrient-populated domain, sampling for chemo-nutrients and existing trails (i.e., regions where the cell had already occupied)^50^. Distinct behaviours, dictating agent movement, reproduction, and elimination, enabled a converging network that solved mazes and found the shortest path between nodes. Tero et al. established a *Physarum* model that leveraged feedback loops between network thickness and protoplasmic flux^20^. Greater streaming between two food sources induced thickening of the connecting network edge. Networks started as randomly meshed lattices, sidestepping initial foraging behaviour, but still evolved to solve transportation problems.

Some flux-based, cellular automatons were established that introduced membrane condition changes and amoebic motion as a basis for network adaptation^14,51^. These models leveraged changes in the “hardness” or “softness” of the cell, analogous to ectoplasmic repolymerization, to establish rules for cytoplasmic flow and morphogenesis, approximating a Steiner-tree and solving a maze. Jones developed both passive^52^ and active^53^ particle-based models for *Physarum* growth. Passive populations refer to those with agents that only respond to their environment, while active populations comprise agents that can also modify their environment. These models captured aspects of both initial network formation (meshing), and adaptive network refinement. Particles moved to approximate nuclei subdivisions throughout *Physarum* growth. Particle resistance was associated with particle density, determined through physical and sensory particle interactions. Following initialization, particles secreted a diffusing chemoattractant, attractive to the nearby particles. This generated a feedback of path reinforcement, as nearby particles were more likely to travel along, and further amplify, well-established trails.

*Physarum* network morphology has also been compared with the Toussaint hierarchy of proximity graphs^54,55^. This hierarchy describes a series of graphs that increase in connectivity, beginning with a minimum spanning tree, where each proceeding graph contains the edges of the former. It has been speculated that *Physarum* grows in stages that mimic successive graphs within the Toussaint hierarchy^54^. Plasmodium sites have been abstracted as a set of points, growing into a nearest-neighbourhood graph, minimum spanning tree, relative neighbourhood graph, Gabriel graph, and finally into a Delaunay triangulation^54,56^. Proximity graphs have also been used to systematically compare *Physarum* networks with urban transport networks: the intersection of a Gabriel graph and *Physarum* ‘graph’ grown between oats representing major Mexican cities was shown to be nearly identical to the intersection of a Gabriel graph and the Mexican motorway graph itself^46^.

In this work, we are concerned with two simplified principles of *Physarum* growth. The first: *Physarum* forages under the influence of attractants across its foraging domain, and establishes a highly granular mesh biased to this attractor field (Fig. 2b,c). The second: *Physarum* refines this granular mesh, establishing from it a simpler network that solves a multi-objective transportation problem (Fig. 2d). This robust biological network balances cost (i.e., total length of all network edges), travel time (i.e., average edge length required to connect two points within a network), and vulnerability (i.e., increase in travel time for the removal of an average segment (i.e., fault) within a network). These constraints are equally ubiquitous in engineered networks across several length-scales.

While some models have considered either foraging and refinement principles exclusively^20,47^, the vast majority have integrated these behavioural elements dependently, cleverly establishing path-reinforcement feedbacks that simulate the sensory, construction, and adaptive refinement behaviours of the biological organism simultaneously^14,50,53^. These systems serve as an exceptional basis for understanding and replicating the feedback mechanism of the *Physarum* cell. However, their intrinsic growth-rule dependencies may also limit external design control and application. A model that describes initial construction independently from network refinement enables compartmentalized system design, which may be particularly beneficial where network refinement constraints are strict (e.g., urban infrastructure). In such a decoupled model, the network refinement process, specifically concerned with balancing the final transport system cost, travel time, and vulnerability to fault, is independent of the preliminary meshing process, introducing stepwise control over network evolution. We hypothesize that by describing construction and refinement behaviours independently, while the constructive feedback mechanism of the organism is sidestepped, a more versatile and accessible network design tool can be established. The objective of this work is to develop and demonstrate this design tool for urban networks.

**Figure 3:**
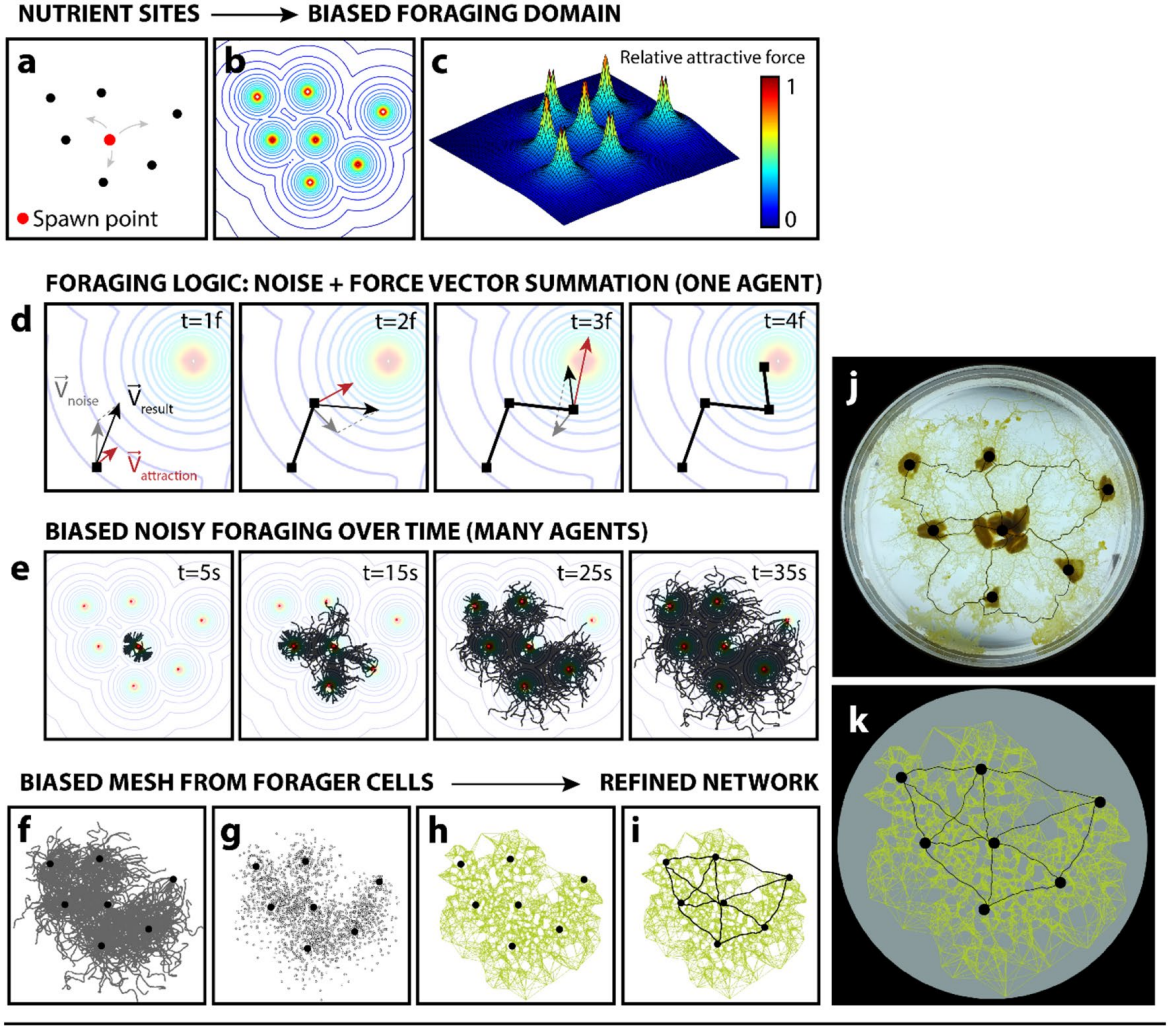
Modelled *Physarum* growth logic. (**a**) Plot showing seven reference food sources. (**b**) Contour plot of the force field generated from (**a**). (**c**) Surface topography showing relative attraction force intensity of (**b**). (**d**) Illustrated foraging logic for a single agent. At each frame, the noise vector is summed with the attraction vector, generated as a function of proximity to food source. (**e**) Screen captures of agent-based model growth over time until all food sources have been colonized. (**f**) Final agent path geometry, from t = 35 s. (**g**) Point cloud from paths in (**f**). (**h**) Mesh generated from points in (**g**). (**i**) Shortest-walk calculation to produce refined network from mesh in (**h**). (**j**–**k**) Biological versus modelled mesh growth with overlaid network for equivalent reference food sources.

**Figure 4:**
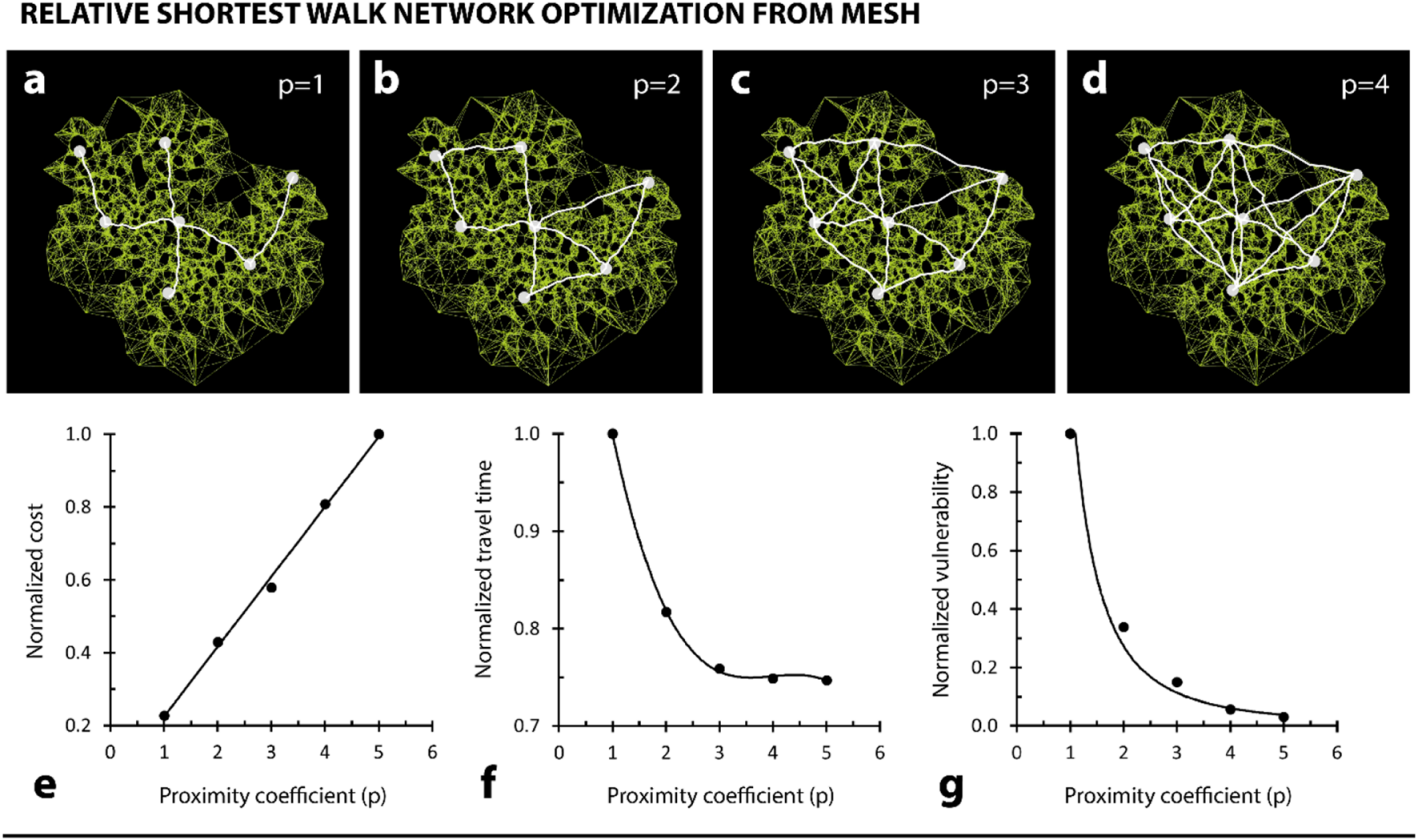
Tunable modelled network behaviour. (**a**–**d**) Different shortest-walk calculations for the same mesh, but with unique proximity coefficient values. (**e**) Plot relating network cost, normalized to the largest cost, to proximity coefficient. Trendline is a linear function. (**f**) Plot relating network travel time, normalized to the largest travel time, to proximity coefficient. Trendline is a third-order polynomial function. (**g**) Plot relating network vulnerability, normalized to the largest travel vulnerability, to proximity coefficient. Trendline is a power function.

**Figure 5:**
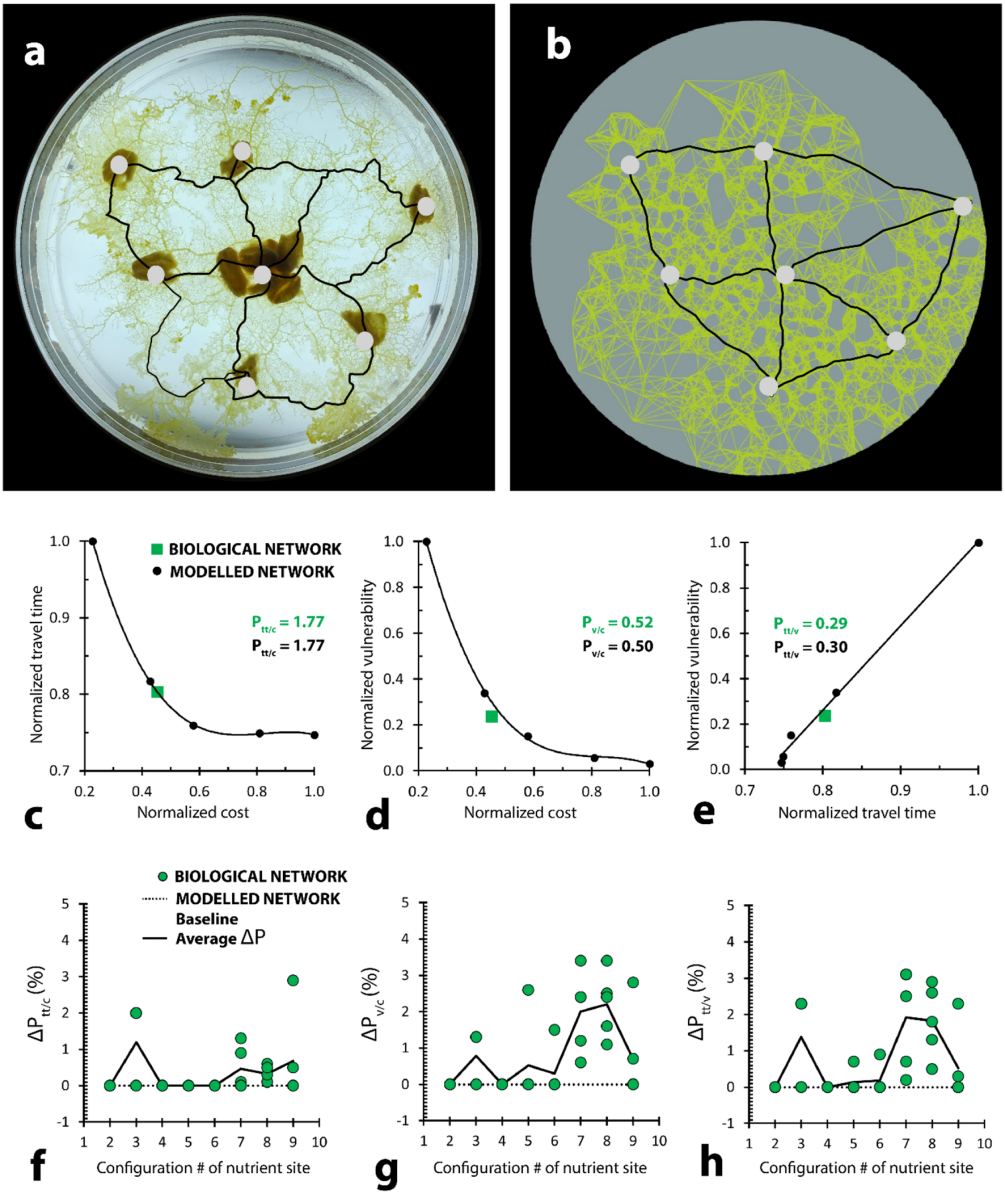
Performance of the modelled networks is within 4% of the performance of the biological networks. (**a**) Grown *Physarum* mesh with overlaid network (t = 5 days). (**b**) Modelled *Physarum* mesh with overlaid network. (**c**) Plot relating network cost to travel time for both grown (green) and modelled (black) networks. Trendline is a third-order polynomial function. (**d**) Plot relating network cost to vulnerability for both grown (green) and modelled (black) networks. Trendline is a third-order polynomial function. (**e**) Plot relating network travel time to vulnerability for both grown (green) and modelled (black) networks. Trendline is a linear function. All performance values normalized to the largest value. Modelled performance values displayed on the plots represent performance for the closest modelled point to the grown point (green) along the trendline. (**f–h**) Performance differences across multiple attractor patterns and growth repeats between biological and modelled networks are consistently within 4% of one another. Green dots represent grown networks. Smooth black lines represent average performance differences between all grown and modelled networks at each oat layout, where difference is taken between the grown network point to the closest point along the modelled network curve. P_tt/c_ is the network travel time over the network cost; P_v/c_ is the network vulnerability over the network cost; P_v/tt_ is the network vulnerability over the network travel time. Plots displayed in c-e represent data from oat layout 7.

**Figure 6:**
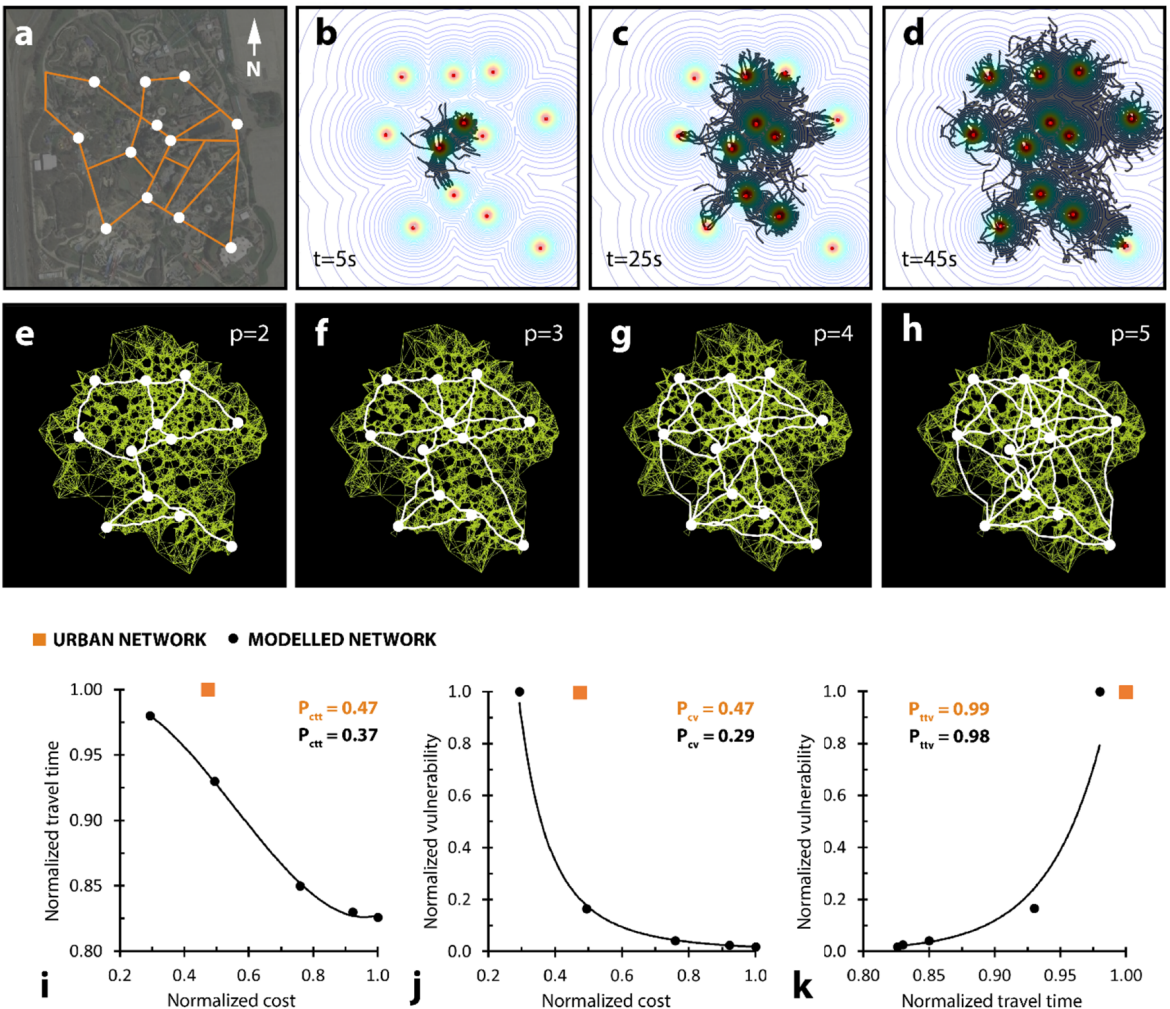
Built versus modelled amusement park network performance is varied. (**a**) Network to describe Canada’s Wonderland. Background image obtained from Google Maps. (**b**–**d**) Modelled growth to establish mesh to describe Canada’s Wonderland. (**e**–**h**) Different shortest-walk calculations for the same mesh, generated from (**d**), but with unique proximity coefficient values. (**i**) Plot relating network cost to travel time for both built (orange) and modelled (black) networks. Trendline is a third-order polynomial function. (**j**) Plot relating network cost to vulnerability for both built (orange) and modelled (black) networks. Trendline is a power function. (**k**) Plot relating network travel time to vulnerability for both built (orange) and modelled (black) networks. Trendline is an exponential function. All performance values normalized to the largest local value. Modelled performance values displayed on the plots represent performance for the closest modelled point to the built point (orange) along the trendline.

**Figure 7:**
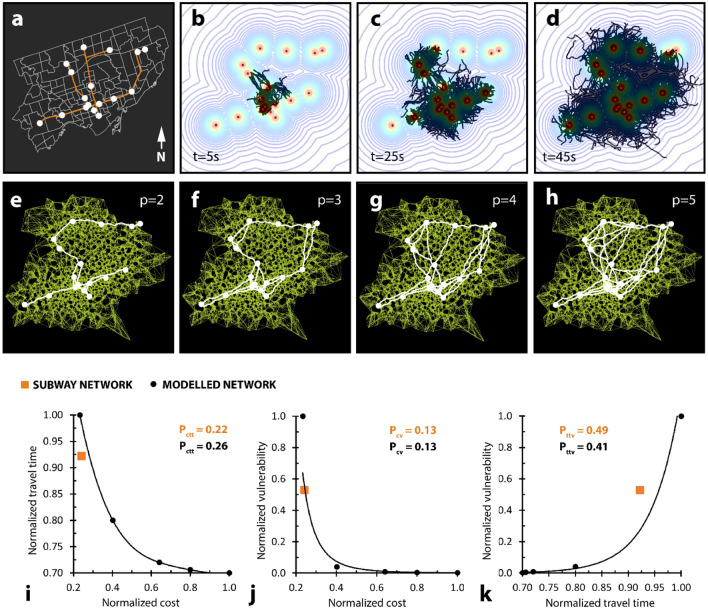
Built versus modelled subway network performance is varied. (**a**) Network to describe Toronto underground subway system. (**b**–**d**) Modelled growth to establish mesh to describe subway system. (**e**–**h**) Different shortest-walk calculations for the same mesh, generated from d, but with unique proximity coefficient values. (**i**) Plot relating network cost to travel time for both built (orange) and modelled (black) networks. Trendline is a fourth-order polynomial function. (**j**) Plot relating network cost to vulnerability for both built (orange) and modelled (black) networks. Trendline is a power function. (**k**) Plot relating network travel time to vulnerability for both built (orange) and modelled (black) networks. Trendline is an exponential function. All performance values normalized to the largest local value. Modelled performance values displayed on the plots represent performance for the closest modelled point to the built point (orange) along the trendline.

Here we report a model that captures, in two discrete steps, the meshing and refining behaviour of the motile *Physarum* cell. We first developed an agent-based simulation to generate a mesh (i.e., proximity graph) uniquely responsive to a given set of food sources (Fig. 3a–h). We next calculated a modified shortest walk between all such sources along this mesh to establish a refined network (Fig. 3i). We demonstrated adaptive control over total walk length (i.e., network cost), travel time, and fault vulnerability (Fig. 4). We demonstrated nearly identical network performance (cost, travel time, fault vulnerability) between our modelled and grown *Physarum* networks (Fig. 5c–e), and we demonstrated varied modelled network performance when compared with existing urban networks (Figs. 6i–k, 7i–k, respectively). Finally, using these techniques that leverage attractor-biased agent propagation, we established urban networks using unconventional sets of attractors—in particular, nodes representative of urban population density (Supplementary Fig. 8a–d).

## Results

### Two-stage growth characterization

We observed the vegetative state (plasmodium) of the common slime mould *Physarum* across a 14-day period (Figs. 1, 2a). We arranged a collection of oats on otherwise nutrient-poor agar plates and placed a small sample of the active plasmodium in the center of each plate. Within the first few days, the *Physarum* established a dense mesh-like lattice, moving towards areas of high nutrient concentration as it foraged (Fig. 2a, t = 2 days). By the third day, the *Physarum* cell began refining this mesh, as it simultaneously continued to expand (Fig. 2a, t = 3 days). Refinement continued into the seventh day (Fig. 2a, t = 7 days), by which time a resolved network became clearly visible. We established three general principles of growth to describe the motile *Physarum* cell: biased foraging, biased meshing, and network refining (Fig. 2b–d). From these, we developed a model to approximate two independent stages that characterized this behaviour: biased meshing (which considers biased foraging) (Fig. 3a–h), and network refining (Figs. 3i, 4).

### Stage one: biased meshing

We implemented an agent-based model to simulate biased foraging and meshing, based on a chemoattractant gradient. We developed a series of simple rules to define agent behaviour. Most fundamentally, the movement of agents was dictated by a summation of two force vectors, one stochastic and the other deterministic. The deterministic component was based on food attraction within a domain of foraging. For a given set of attractor points (oats), we established an attractor field within the domain of *Physarum* foraging. We defined the square of the force exerted by a food source on any given agent to be inversely proportional to the distance between the agent and food source. Equation (1) describes the relationship between the magnitude of the force felt on a given agent (*F*_*food on agent*_), the distance between that agent and a closest nutrient source (oat flake) (*D*), and a force constant (*c*). This force constant, *c*, can be specified in the model to alter the noisiness of foraging behaviour. In our experiments, we modelled *Physarum* with a force constant of 10. For a set of oats, as digitized from photographs of experimental *Physarum* growth (Fig. 3a), the relative attractor field is illustrated in Fig. 3b,c. This field specifies the attraction force felt by an agent given its position within the domain. Distances between agents and each attractor point were continuously sampled, and the attractive force of a closest food source was mapped to each agent.

1 $${F}_{food \; on \; agent}= \sqrt{c/D}.$$

We initialized modelled *Physarum* growth by first generating a population of agents, *n*, where *n* = 50 for all experiments, from the center of the substrate domain (Fig. 3d, t = 5 s, and at the spawn point demonstrated in Fig. 3a). The substrate was defined to be equiaxial, measuring 800 × 800 pixels. Agents foraged at a constant rate (1.5 pixels per frame). We captured growth at 60 frames per second (agents therefore foraged at 90 pixels per second). Each frame, we generated the heading component of the stochastic movement vector using a one-dimensional Perlin noise (number-generating) algorithm. Perlin noise is an algorithm initially developed for procedural texture generation in computer graphics^57^. This algorithm maintains a “memory” of its latest state, and produces a number that is related to the number generated directly before it. The Perlin noise algorithm generates numbers within a specified range at a specified time-step, known as the first octave. To produce smoothed out pseudo-random behaviour, the function sums the set of numbers produced in this first octave with those produced at all of the smaller octaves, where each subsequent octave doubles the frequency of the timestep and halves the range. This approach enables a time-dependent number generation, producing a smoother pseudo-random walk. We used Perlin noise to generate the x and y directional components of the stochastic vector for every agent at every timestep.

At each frame, this stochastic heading vector was summed with the deterministic vector corresponding to the force exerted by the nearest food source to each agent (i.e., both magnitude and directional components were summed) (Fig. 3d). As the magnitude of the deterministic force vector increased with food proximity (Eq. 1), agents foraged more deterministically towards a given food source as they approached that given food source. This is illustrated in Fig. 3d, as the attraction vector (red) grows proportionally larger, and becomes proportionally more dominant compared to the stochastic “noise” vector (grey), from t = 1f to t = 3f.

Velocity magnitude was reset directly after vector summation. This enabled a constant *Physarum* growth rate, but accounted for differences in force magnitude when calculating agent heading through vector summation (these differences in vector magnitude are clearly demonstrated in Fig. 3d). Food sources attracted agents and became colonized. Figure 3 demonstrates a clustering effect around each food source, over time. Once a given food source was depleted (we defined this event once *n*/10 agents reached that food source), it became no longer attractive to the *Physarum*. Instead, the depleted food source became a source of offspring (providing energy for further growth), and produced a new population of *n* agents, which foraged in the same manner as described above.

Once all food sources were depleted (i.e., once all food sources were reached by *n*/10 agents), we terminated the particle-growing phase of the simulation (Fig. 3e, t = 35 s). Figure 3f illustrates the agent foraging trails throughout the complete growth process. We obtained a computationally inexpensive description of this cellular morphology by printing the locations of these agents (as points, analogous to differentiated *Physarum* nuclei) at a stepped frame rate over the duration of the simulation (one point per three frames for all simulations) (Fig. 3g). From this cloud of nuclei points, we generated a mesh-like proximity-graph (Fig. 3h). A proximity graph comprises a set of points (in our case, both the food sources and nuclei trail locations) and a set of edge links that describe the proximity between all points. Here, we established the proximity graph in Fig. 3h by first searching for two-dimensional proximity between point pairs in Fig. 3g. From this information (i.e., a distance matrix for all pairs of points), we connected each point to its *p*_*m*_ closest neighbours (where *p*_*m*_ = 10 neighbours for all simulations). Here, *p*_*m*_ refers to the number of neighbours to which each point was connected. What we refer to throughout this paper as our site-responsive biased mesh (Fig. 3h) can therefore also be defined as a 10-neighbour proximity graph. The effect of *p*_*m*_ on the density and cost of the mesh is demonstrated in Supplementary Fig. S2. The density (i.e., number of linkages) in the mesh increased with *p*_*m*_ (Supplementary Fig. S2a). The total mesh cost also increased with *p*_*m*_ (Supplementary Fig. S2b). This increase was determined to be linear (Supplementary Fig. S2b, linear trendline).

### Stage two: adaptive network refining

To produce a refined network (Fig. 3i), we calculated a modified shortest-walk along the proximity graph (mesh) produced in Fig. 3h. A shortest-walk network represents the most cost-effective (i.e., shortest) network that connects a set of points (food sources) to a certain number of its neighbours through a given set of edges. We first searched for two-dimensional proximity between food source pairs in order to generate a distance matrix that stored the distances between every pair of food sources. By defining our biased mesh produced in Fig. 3h as the calculation domain (i.e., set of possible edges) for the shortest-walk, we produced a modified shortest-walk network to connect all food sources. This network was visually comparable to a network connecting equivalent points grown biologically (Fig. 3j,k). The network demonstrated in Fig. 3k, for example, represents the shortest-walk network that connects each food source to a minimum of three closest neighbours. In this case, the network in Fig. 3k can be achieved with thirteen distinct edges between food sources. The networks produced biologically and computationally, as demonstrated in Fig. 3j,k respectively, are compared in more detail in Fig. 5, and discussed below.

For the same set of points used to grow and computationally mesh an arbitrary network (Fig. 3a), we demonstrated adaptive refinement control over our modelled network by establishing a proximity coefficient, *p*. Here, *p* is used to define the number of closet neighbours to which each food source will connect, when a shortest-walk is calculated. We demonstrated an increase in network connectivity with an increase in *p* (i.e., more edge connections) (Fig. 4a–d). Based on network definitions given by Tero et al.^20^, we established three descriptions for network efficiency, and we demonstrated the effect of *p* on each (Fig. 4e–g). We defined cost as the total length of all edges within a network. We defined travel time as the average edge length required to connect two points within a network. And we defined vulnerability as the increase in travel time for the removal of an average segment (i.e., fault) within a network. We computed this increase by systematically removing each element from the network, calculating for each removal the average increase in network travel time. If two points became disconnected through this calculation, we defined the distance between points as the length of the total network. We demonstrated that network cost increased linearly (linear trendline) for an increase in proximity coefficient (Fig. 4e). We also demonstrated that this trend was independent of the network—obtaining an identical relationship for networks generated based off three randomly-generated food source sets (where the number of food source points was set to 5, 10, and 50) (Supplementary Fig. S4). Finally, we demonstrated that both network travel time and vulnerability decreased with an increase in proximity coefficient (Fig. 4f,g). The former trend is described with a third-order polynomial (Fig. 4f), and the latter trend with a power function (Fig. 4g).

### Effect of force constant on foraging morphology and network refinement

Supplementary Fig. S5a illustrates the effect of force constant strength on agent trail morphology. Here, ten simulations were run (*n* = 50), each with a different force constant, ranging from *c* = 1 to *c* = 1000. The relative force vector strength increased with the force constant (Eq. 1). Therefore, the relative effect of the force vector, when summed with the Perlin noise vector, also increased with the force constant. This resulted in a change in the noisiness of foraging: as the force constant increased, the agents moved more deterministically and uniformly—in the direction of the force vector (Supplementary Fig. S5a). The total trail cost (i.e., the number of agents in the foraging domain multiplied by the runtime of the simulation) was largely independent of the force coefficient (Supplementary Fig. S5a). The total mesh cost (described above), however, was indirectly affected by the force constant. With a smaller force constant, trail generation was more distributed, requiring a high mesh cost to obtain appropriate coverage (Supplementary Fig. S5c). The mesh cost displayed asymptotic behaviour as a function of force coefficient (Supplementary Fig. S5c). Due to this effect, the shortest-walk (i.e., finalized network) cost was also indirectly impacted by force coefficient. More elaborate, less cost-effective, meshes were more connected, and provided a greater calculation domain to perform a shortest-walk. Therefore, opposite the effect on mesh cost, shortest-walk cost increased linearly with force coefficient (Supplementary Fig. S5d).

*Physarum* forages both with a stochastic and sensory (i.e., deterministic) component. These are analogous, in our model, to the Perlin noise and attractive force vector components, respectively—which we sum. The relative magnitude of these components determines the overall distribution of the initial foraging mesh. For example, when the force constant was low (Supplementary Fig. S5a, c = 1), the stochastic (Perlin noise) vector was dominant in determining the agent foraging direction, resulting in a noisy forage. Oppositely, when the force constant was high (Supplementary Fig. S5a, c = 1000), the attractive force vector was dominant in determining the agent foraging direction, resulting in a deterministic forage (here agents foraged in the direction of the nearest food source uninterruptedly). The fraction of the foraging domain accessible (i.e., within a certain number of pixels) to the agent trail decreased as a function of the force coefficient (Supplementary Fig. S5e–g). This was demonstrated for three accessibility ranges: corresponding to within 20% of the total domain width (Supplementary Fig. S5e), within 10% of the total domain width (Supplementary Fig. S5f), and within 1% of the total domain width (Supplementary Fig. S5g). We chose to perform all simulations with a force constant of 10, as we likened the model foraging behaviour at this force constant to the foraging behaviour of the biological organism (Supplementary Fig. S5a, c = 10).

### Effect of starting node choice on network morphology

Our multiphase model allows for high variability in the preliminary mesh with no net impact on final network morphology. The meshing stage produces an extensive domain of trail samples for refinement, allowing for course corrections between stages. Supplementary Fig. S6a shows seven independent meshes, developed by initializing the simulation of *Physarum* growth from each of the seven unique attractor points. As can be seen, the morphology of the preliminary meshes varies extensively. However, following the model’s network refinement stage, we see that network morphology (i.e., path segment similarity) is consistent across simulation iterations. This is confirmed quantitatively, as we see less than 1% variation in cost between each of the seven networks, validating that foraging can be initialized from any start point with no effect on final network morphology.

### Model validation against biology

To compare modelled with biological growth, we conceptualized eight unique spatial arrangements of nutrient sites (oats) atop an agar plate, and grew *Physarum* networks between them. Layouts comprised a unique number of oats (ranging between 2 and 9), and, to test for variation in growth behaviour, we repeated growth for each arrangement five times. We obtained a matrix of 40 unique networks corresponding to the eight predesigned spatial attractor layouts (Supplementary Fig. S7a). We then digitized the precise attractor layouts and simulated growth using our model. For consistency, we simulated *Physarum* growth five times for each of the eight attractor layouts, obtaining a comparable matrix of 40 unique modelled networks.

To compare the biological and modelled *Physarum* networks, we first developed a methodology for simplifying biological structures into refined networks. We identified the largest, most relevant veins within grown networks by binarizing photographs taken of their form after five days, replacing each pixel (valued between 0 and 255) with either a black (255) or white (0) pixel value. We set a threshold for black–white pixel differentiation at a pixel value of 67. This segmentation process filtered out smaller veins, and revealed the largest vein structures, which were used to define the biological network for quantitative comparison. For accurate performance characterization and comparison, we simplified biologically-grown network geometries by fitting smooth lines along jagged paths to eliminate foraging noise. We also neglected paths that ventured outside of the relevant foraging boundary (i.e., towards the petri dish wall), and filtered out the more costly of two redundant paths. This network digitization process is exemplified in Supplementary Fig. S7. Figure 5a,b compares a grown and modelled network for one particular set of food sources with seven oats/attractor points.

To compare networks using the defined parameters of cost, travel time, and vulnerability, we defined three general performance values, P_tt/c_, P_v/c_, and P_v/tt_, corresponding to the normalized performance ratio between the travel time and cost, the vulnerability and cost, and the vulnerability and travel time, respectively. Modelled performance values are displayed in Fig. 5c–e as a best fit curve (trendline) between network results generated with proximity coefficients between 1 and 5, while biological performance values are displayed as a point. For the particular set of oats demonstrated in Fig. 5a,b, the grown *Physarum* cell performed similarly to our model, falling within 1%, 4%, and 4% of the graphed trendline for P_tt/c_, P_v/c_, and P_v/tt_, respectively (Fig. 5c–e): average network travel time per cost for the *Physarum* was identical to the closest point along the modelled trend (1.77 versus 1.77) (Fig. 5c); average network vulnerability per cost for the *Physarum* was within 4% of the closest point along the modelled trend (0.52 versus 0.50) (Fig. 5d); and average network travel time per vulnerability for the *Physarum* was within 4% of the closest point along the modelled trend (0.29 versus 0.30) (Fig. 5e). It was also observed that network morphology differences between networks modelled along the same layout of oats were negligible, and within 1% of the cost, travel time, and vulnerability of one another.

More broadly, we compared P_tt/c_, P_v/c_, and P_v/tt_ for biological networks grown across all eight oat layouts to our model’s corresponding performance curve (generated using 5 modelled networks, each with a different proximity coefficient, illustrated in Supplementary Fig. S7b). P_tt/c_, P_v/c_, and P_v/tt_ for each of the five grown networks were compared to the closet point along the modelled curve. The differences in P_tt/c_, P_v/c_, and P_v/tt_ between grown and modelled networks are shown in Fig. 5f–h, respectively. Despite observable trends in morphology differences between grown and modelled networks (e.g., n = 3 in Supplementary Fig. S7), performance discrepancy values never exceeded 4%. This result is partly explained by performance metric dependence, where more costly networks tended to have lower travel times and vulnerabilities, while cheaper networks tended to have higher travel times and vulnerabilities. In 55%, (22/40) of the growth iterations, grown networks were directly identical to a modelled counterpart (such a case results in a 0% difference in P_tt/c_, P_v/c_, and P_v/tt_, and is particularly common for n = 2, 4–6). The remaining grown networks performed, on average, within 0.4%, 0.8%, and 0.7% of the P_tt/c_, P_v/c_, and P_v/tt_ curves of their modelled equivalents, respectively, confirming broad agreement between systems.

### Model comparison to urban infrastructure

We used our model to generate networks that describe two existing systems of urban infrastructure at two scales: an amusement park (Canada’s Wonderland in Toronto, Ontario), and a subway system (Toronto Transit Corporation Subway system in Toronto, Canada). From an aerial photograph of Canada’s Wonderland, we digitized selected attraction points (food sources) and edge connections (network) (Fig. 6a). We used these digitized attractor points to generate a nutrient domain to model *Physarum* growth (Fig. 6b–d). We produced several network iterations, for unique proximity coefficient values (Fig. 6e–h). We compared modelled with built network performance (Fig. 6i–k). Our modelled network performed more favourably over the existing network, displaying 27% lower P_ctt_ (cost × travel time) (0.37 versus 0.47) (Fig. 6i), 61% lower P_cv_ (cost × vulnerability) (0.29 versus 0.47) (Fig. 6j), and 2% lower P_ttv_ (travel time × vulnerability) (0.99 versus 0.98) (Fig. 6k). As before, modelled P_ctt_, P_cv_, and P_ttv_ values were calculated from the closest point to the urban network along the modelled trendline.

We obtained spatial data of the four subway lines and 74 stations that make up the underground subway network in Toronto, Canada, and we selected seventeen core stations to represent network attractors (Fig. 7a). We modelled a network to represent *Physarum* growth across the seventeen core subway stations (Fig. 7b–h), and compared modelled with built network performance (Fig. 7i–k). Our modelled network performed differently compared to the existing subway system, displaying 14% higher P_ctt_ (cost × travel time) (0.26 versus 0.22) (Fig. 7i), identical P_cv_ (cost × vulnerability) (0.13 versus 0.13) (Fig. 7j), and 19% lower P_ttv_ (travel time × vulnerability) (0.41 versus 0.49) (Fig. 7k). As before, modelled P_ctt_, P_cv_, and P_ttv_ values were calculated from the closest point to the urban network along the modelled trendline.

### Population density-defined attractor network

**Figure 8:**
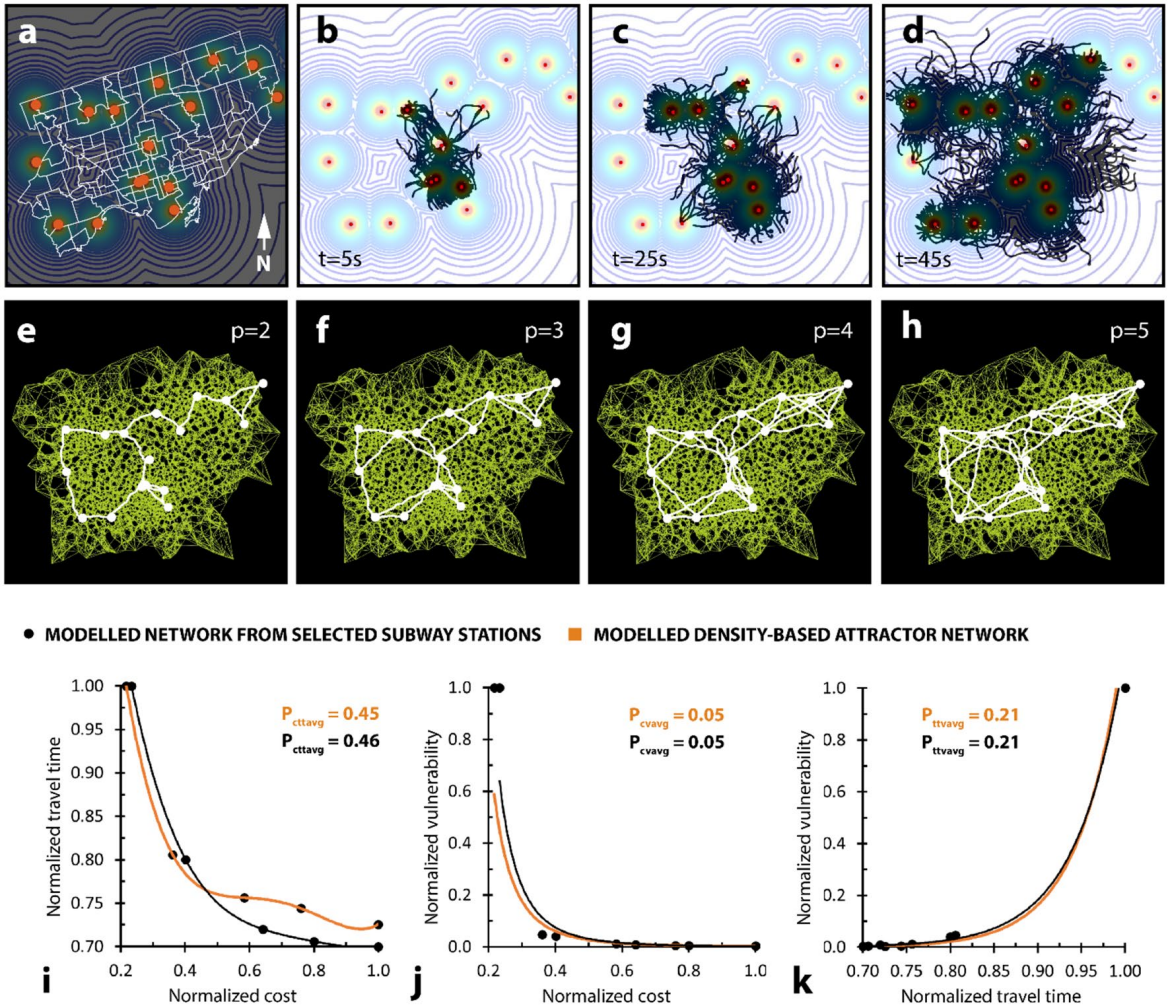
Comparison between modelled networks generated with population-density- and subway-station-defined attractor points. (**a**) Dot density population distribution of city of Toronto, where each dot represents 150,000 residents (**b**–**d**) Modelled growth to establish mesh to describe hypothetical subway system, based off attractors generated in a. (**e–h**) Different shortest-walk calculations for the same mesh, generated from d, but with unique proximity coefficient values. (**i**) Plot relating network cost to travel time for both density-based network model (orange) and station-based network model (black). Trendlines are both fourth-order polynomial functions. (**j**) Plot relating network cost to vulnerability for both density-based network model (orange) and station-based network model (black). Trendlines are both power functions. (**k**) Plot relating network travel time to vulnerability for both density-based network model (orange) and station-based network model (black). Trendlines are both exponential functions. All performance values normalized to the largest local value. Modelled average performance values displayed on the plots represent average performance values cross all network iterations.

We demonstrated that networks can be generated not only from predefined points of interest, but from various forms of spatial data that are interpretable as a set of attractor points. We used population data for neighbourhoods in Toronto, Canada to generate seventeen attractor points that represented the population distribution of the city (i.e., each point represents 150,000 residents) (Fig. 8a). We simulated *Physarum* growth across this set of points (Fig. 8b–d), and generated alternative subway networks for unique proximity values (Fig. 8e–h). We compared network performance between density-generated attractors and station-generated attractors (Fig. 8i–k). Modelled performance was nearly identical across the two systems. The population density-defined network displayed 2% lower P_cttavg_ (average cost × travel time) (0.45 versus 0.46) (Fig. 8i), 5% lower P_cvavg_ (average cost × vulnerability) (495e−4 versus 524e−4) (Fig. 8j), and 1% higher P_ttvavg_ (average travel time × vulnerability) (211e−3 versus 209e−3) (Fig. 8k).

## Discussion

We implemented a discrete, two-phase *Physarum* model that leveraged independent mechanisms to generate both a mesh and final network responsive to the attractor field in which they were established. We demonstrated that this biologically-inspired model, despite omitting the biological constructive feedback mechanism that enables growth, could accurately describe plasmodial *Physarum* growth to within 4% and, moreover, that it could be leveraged more directly as a simple tool to design networks comparable to existing systems of urban infrastructure.

We conclude that network performance, defined using cost, travel time, and vulnerability, between our grown and modelled *Physarum* systems was highly similar (within 4% across various attractor layouts and growth iterations). We also conclude that network performance varied between our modelled *Physarum* system and existing urban infrastructure systems. The discrepancies in this performance can prove to be highly informative, serving to illuminate strengths, susceptibilities, and overarching design differences between biological and anthropic networking systems. For example, our bio-inspired model performed superiorly in all measurable categories compared to Canada’s Wonderland. For an equivalent cost, we generated a network that was over 80% less vulnerable (Fig. 6j) and almost 10% less time-consuming (Fig. 6i) than the existing system. This difference may be because amusements parks have different design goals to conventional transportation systems—where excess costs and travel times may be considered favourable (e.g., a greater path network to land area ratio may increase consumer opportunities). Our bio-inspired model performed less favourably in some measurable categories compared to a more conventional transportation system—Toronto’s underground subway network. For an equivalent cost, we generated a network that was identically vulnerable to fault (Fig. 7j), but almost 10% more time-consuming (Fig. 7i) than the existing subway system. For an equivalent travel time, however, we generated a network that was about 40% less vulnerable to fault than the existing subway system (Fig. 7k). Biological organisms are characteristically resilient, able to regenerate and adapt to faults. This model may serve as a basis for more resilient network construction—which is becoming increasingly relevant with the unpredictable impacts of climate change.

When comparing grown and modelled networks, graphs in Fig. 5g,h demonstrate two small peaks in performance difference occurring at n = 3 and between n = [6, 9]. The discrepancies between layouts n = [6, 9] can most likely be explained by the increasing complexity of the grown networks and greater range in decision making variability of the organism. The discrepancies between networks grown within the attractor layout of n = 3 (triangle shape), on the other hand, may point to an important limitation in the model. In 3 of 5 growth iterations, the organism formed a Steiner minimum tree between oats, a result that differs from the complete or near-complete triangle morphologies produced by our model. This discrepancy emphasizes the limited number of discrete proximity coefficient steps the model can choose between, unable to find optimized network solutions using paths that do not directly link two attractor points. Such a limitation is less important as networks increase in complexity, but may bound efficient growth to a minimum oat layout of n = 4.

Compared to other models, our work enables multi-objective (e.g., cost, travel time, vulnerability) network design, independent of preliminary growth. Networking systems across various scales, built for various environmental and functional regimes, must also adhere to complex and competing constraints, and may also benefit from late-stage, multi-objective design control. Relative to existing models, our stepwise algorithm omits critical feedback loops of streaming behaviour, limiting its effectiveness capturing the characteristics and morphological dynamics of *Physarum* growth. Instead, our model emphasizes broad applicability: compared to nearly all current models, which do not allow for stepwise interruption and tuning, our work offers an opportunity for designer feedback and input amidst the growth process, enabling new avenues for applied network generation. Additionally, our model allows for flexibility in starting condition. Diverging from the behaviour of live *Physarum*, and the many models that draw from it, our algorithm demonstrates consistent network results independent of the point from which growth was initialized. We therefore imagine that the presented model will prove especially useful for applications where design objectives are strict (e.g., in situations where start node cannot be controlled, or where more ‘biologically-accurate’ *Physarum* models cannot produce network morphologies within a tight application domain).

Our model specifically enables network growth from attractor-based information. Networks can be established through simple data translation (e.g., population distribution as attractors, information density distribution as attractors, physical boundaries as anti-attractors) from the urban to the microelectronic network scale. Importantly, we note that our model currently functions in ‘idealized’ environments with attractors, and does not include repellants (anti-attractors). It is not clear how *Physarum* negotiates environments where both attractors and anti-attractors are present. For instance, many of the complex behaviours exhibited by *Physarum* cannot be explained by simple stimulus–response models^58^. In one set of physical experiments, when *Physarum* was introduced to bi-modal stimuli—consisting of a standard mixture of both attractor and anti-attractor materials—the plasmodium varied its decision-making and network morphologies. For the same attractor concentration, the organism was inconsistently attracted to the stimuli, at times even completely avoiding the stimuli^58^. Developing more advanced models that can function in environments with both attractors and anti-attractors would be a be a beneficial future endeavour.

Additionally, from our existing proximity graph model, it will be easy to develop networks that adapt their path segment capacities depending on usage. Path segments can take on diameters proportional to their use frequency, where frequency can be determined while calculating shortest network travel times between point pairs. This added dimension of network morphology would enable more broad applicability—to solve flux-based and path-planning problems simultaneously.

Perhaps most fundamentally, this work provides a simple baseline for developing and quantitatively assessing network performance at the urban scale. Infrastructural networks are too often constructed without standard and quantitative design principles, and the model described here can help benchmark both existing and future network developments towards achieving more efficient and resilient cities. Additionally, we note that the stepwise principles of growth introduced here may find relevant applications in other network problems across multiple length-scales, representing exciting trajectories for future inquiry.

## Conclusions

*Physarum* can operate as a biological computer, and is particularly useful for solving anthropically-shared problems of transport network design. We characterized *Physarum* growth as a two-phase process (biased meshing and network refining), and described such behaviour with a two-phase digital model. Our model produced networks that performed nearly identically to the biological organism, but differently to existing urban infrastructure. This model enables tunable, multi-objective network design, independent of the environmental regime. As such, it can serve as an urban design tool that offers biologically-informed rules for network construction.

## Materials and methods

### Sample preparation and imaging

*Physarum polycephalum* (Carolina Biological Supply) was grown on 1.5% (weight/volume) nutrient-free agar (BioShop), prepared in a 9-cm-diameter polystyrene petri dish (VWR). Oat flakes (Carolina Biological Supply) were placed across the substrate in a predesigned pattern, and the active plasmodium (approx. 2-mm-diameter sphere) was placed atop a prespecified oat. Cardstock cutouts were used to ensure consistent oat placement for repeated experiments. Additional oat flakes were placed around the plasmodium to accelerate growth. Samples were stored in low-light, room-temperature conditions, except during imaging. Samples were imaged daily with a Nikon D3200 DSLR camera, and illuminated from below (Fig. 2a). Additional imaging was obtained after 2, 4, 7, and 12 days, using a BW500 digital microscope (Fig. 1m–o, Supplementary Fig. S1e,f) and AmScope MU1000 microscope digital camera (Fig. 1c–j).

### Image digitization

We converted digital photographs, taken of the organism and substrate after 5 and 7 days, into points (attractor points) and vectors (network edges). Images were analyzed using ImageJ software (National Institutes of Health. Bethesda, MD, United States), and vectorized in Adobe Illustrator (Adobe. San Jose, CA, United States). For identifying relevant paths from network photographs, images were binarized using a specified black–white pixel value threshold in ImageJ. Point data was used to establish input attractor point information. Vectorization was also used to interpret urban infrastructural networks (Fig. 5a).

### Model development

We developed an agent-based model in Processing (M.I.T. Cambridge, MA, United States). Agent locations, stored as points, were exported into the modelling environment Rhinoceros (Robert McNeel & Associates. Seattle, WA, United States). All network refinement (shortest-walk calculations) and network analysis was done in Grasshopper, a visual coding plugin for Rhinoceros (Robert McNeel & Associates. Seattle, WA, United States). We used Grasshopper to translate modelled network segments into simplified linework, for more efficient network assessment.

### Data acquisition

Spatial transportation and population data was obtained from the Toronto Police Services (City of Toronto Open Data Portal). Attractor points that represented population density were calculated as dot density data points in ArcMap (Esri. Redlands, CA, United States).

## Supplementary Information

Supplementary Figures.

## Data Availability

No datasets were generated or analyzed during the current study. All code can be made available by the corresponding authors upon request.
