# Supplementary material for: Stepwise slime mould growth as a template for urban design
Source: Sci Rep. 2022 Jan 25;12:1322. doi: 10.1038/s41598-022-05439-w (PMC8789834; doi:10.1038/s41598-022-05439-w)
Supplement: Supplementary file 1 — Supplementary Figures. [file 41598_2022_5439_MOESM1_ESM.docx]

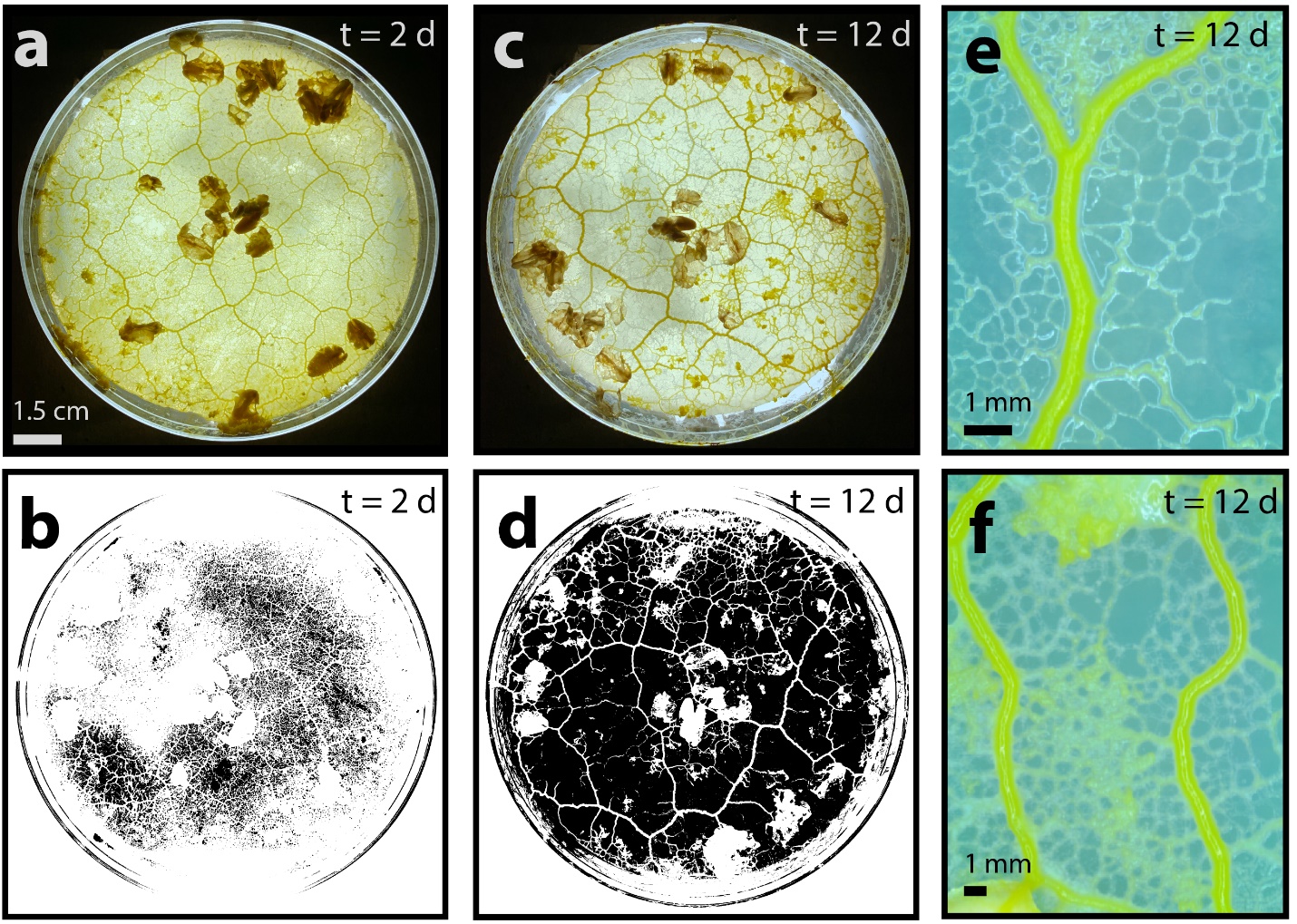


**Figure S1. Network refinement over time.** (**a**) Image of *Physarum* cell after 2 days of meshing. (**b**) Binarized image of (a), with consistent greyscale threshold, to demonstrate density of mesh (white). (**c**) Image of *Physarum* cell after 12 days of meshing. (**d**) Binarized image of (c), with consistent greyscale threshold, to demonstrate refinement of mesh (white). (**e-f**) Images of *Physarum* cell that demonstrate differential path refinement after 12 days of growth.


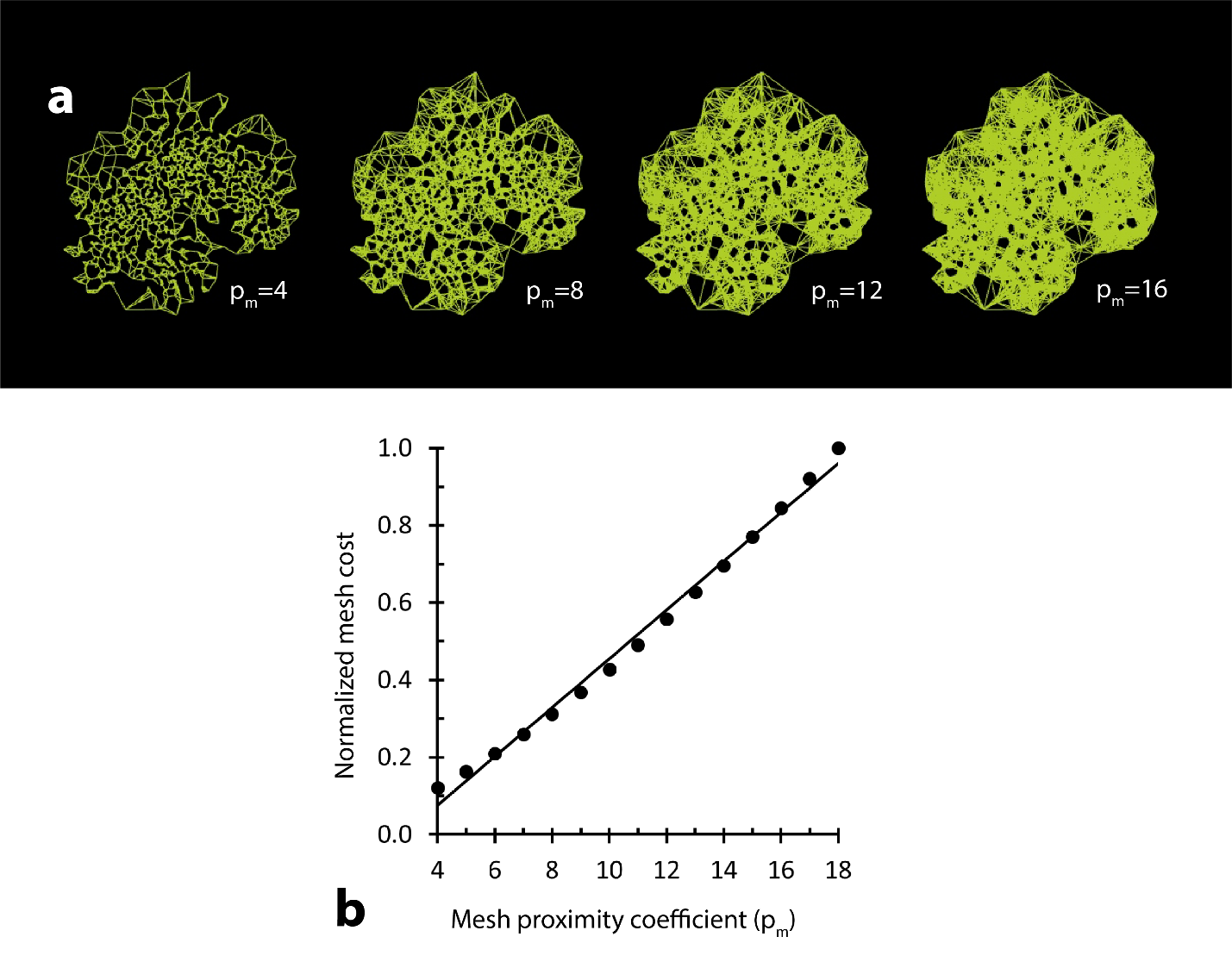


**Figure S2. Control over mesh density and cost.** (**a**) Modelled mesh (proximity graph) increases in density as the number of points to which each mesh point is connected (p_m_) increases. (**b**) Mesh cost increases as a function of p_m_. Trendline is linear. Mesh cost is normalized to the largest cost.


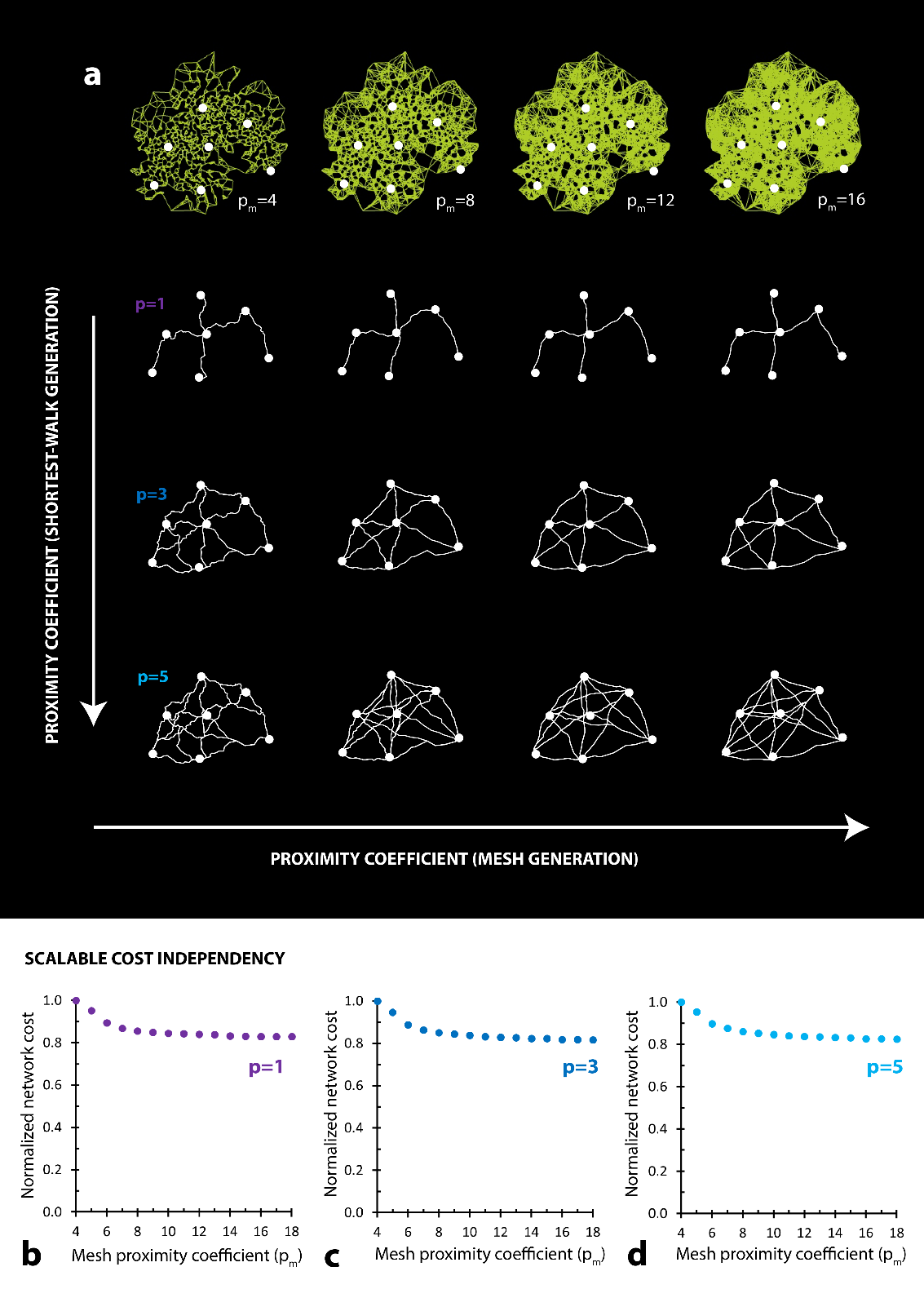


**Figure S3. Effect of mesh proximity coefficient (p_m_) on final network.** (**a**) First row: modelled mesh (proximity graph) for four definitions of proximity (p_m_). Second row: corresponding shortest-walk networks for p = 1. Third row: corresponding shortest-walk networks for p = 3. Fourth row: corresponding shortest-walk networks for p = 5. (**b-d**) Network cost as a function of mesh proximity coefficient (p_m_) is independent of network proximity coefficient (p). Costs are normalized to the largest local values in each case.


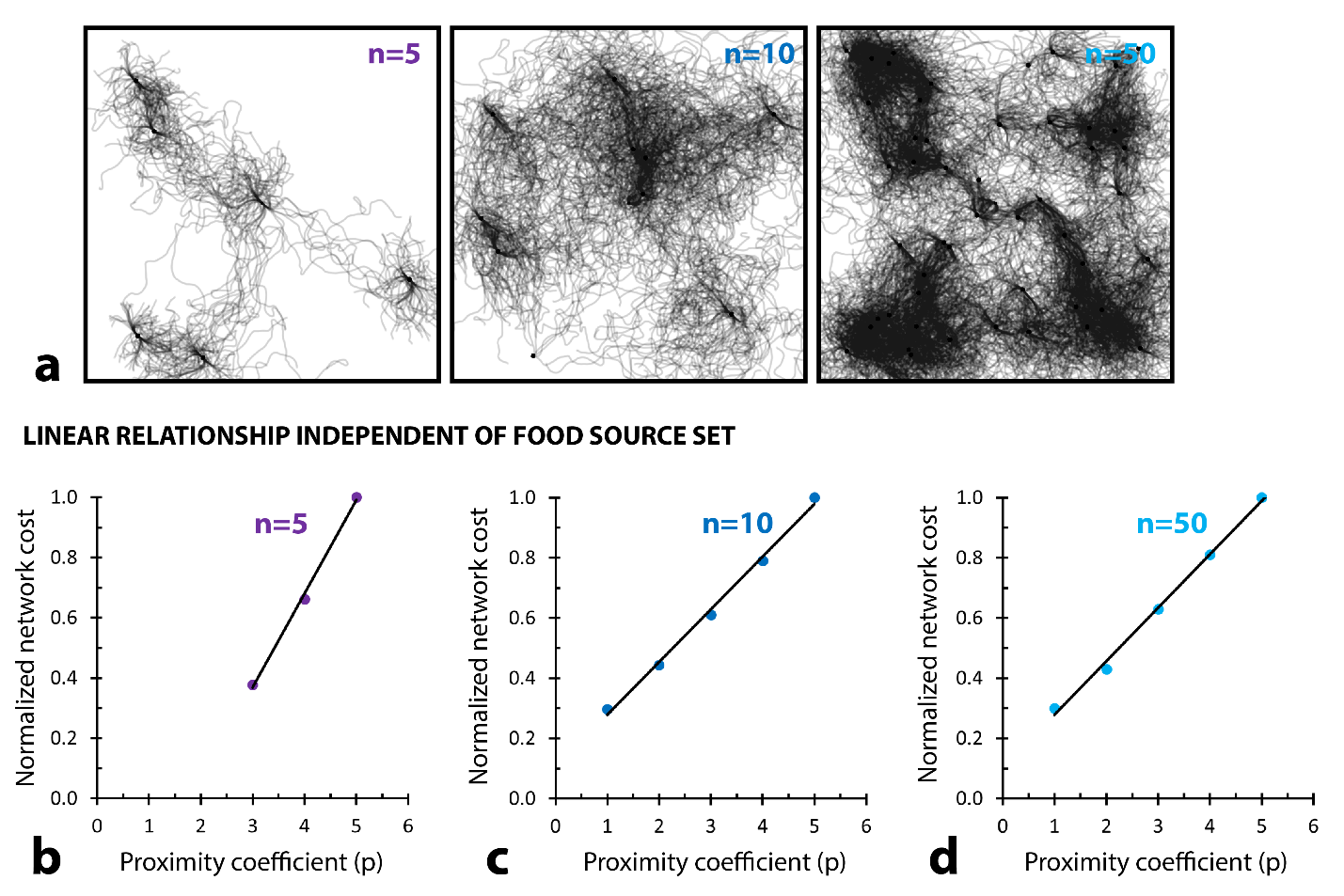


**Figure S4. Network cost varies linearly with network proximity coefficient independent of network size or morphology.** (**a**) Simulation captures of modelled agent trails for three randomly-generated sets of food sources (n = 5, 10, 50). Images taken after all food sources were depleted. (**b-d**) Network cost increases linearly with proximity coefficient. This relationship is independent of the network, however the slope is varied across networks. Costs are normalized to the largest local values in each case.


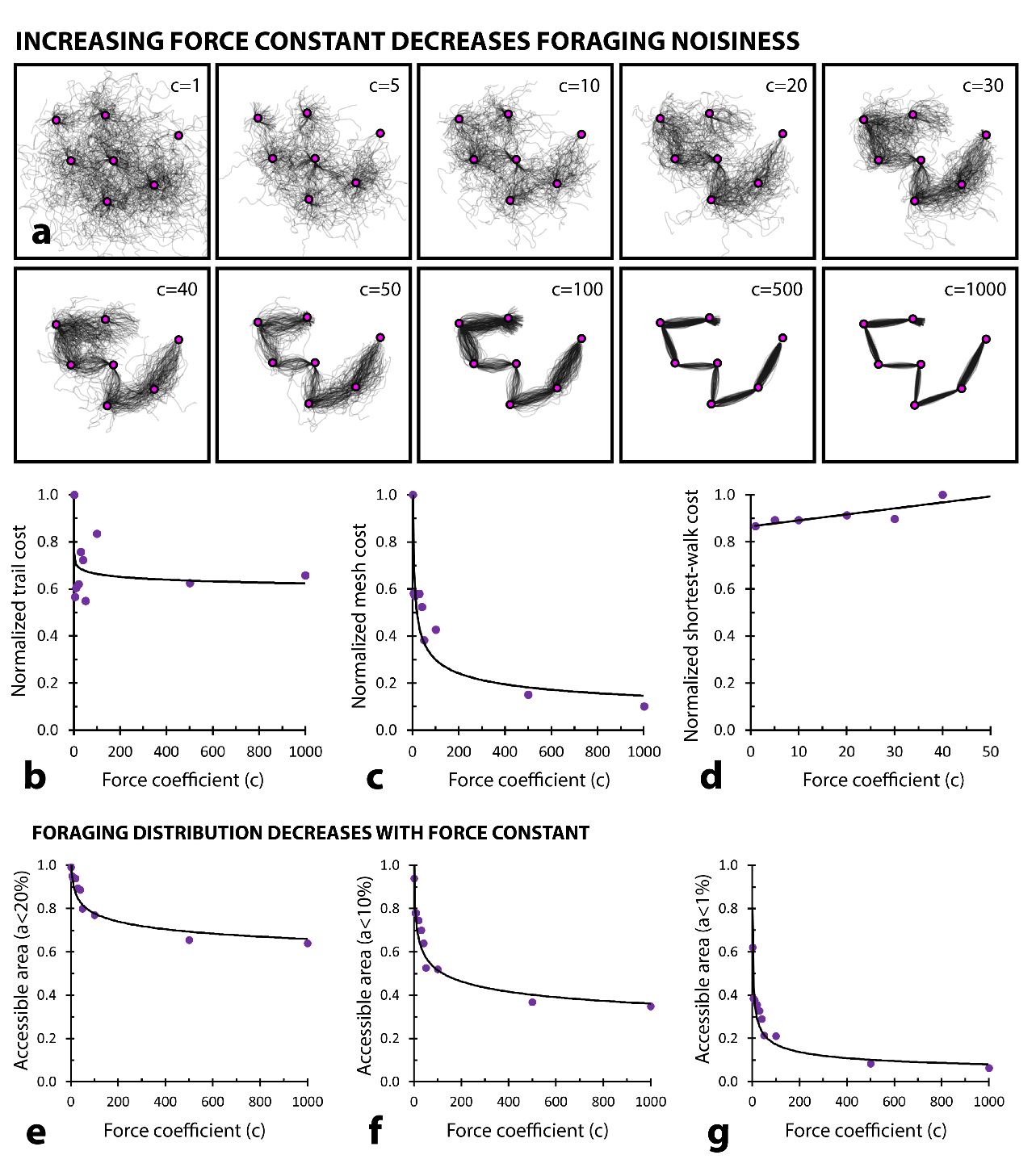


**Figure S5. Effect of force constant (*c*) on modelled agent foraging behaviour.** (**a**) As the force constant increases, so too does the stochastic nature of the foraging agents. (**b**) Normalized trail cost as a function of *c*. Trail cost is generally independent of *c*. (**c**) Normalized mesh cost as a function of *c*. Mesh cost decreases as a function of *c*. (**d**) Normalized shortest-walk (final network) cost as a function of *c*. Network cost gradually increases as a function of *c*. (**e-g**) The fraction of the foraging domain accessed, and therefore accessible, to the modelled *Physarum* agents decreases as a function of *c*. This fraction is defined as the foraging area accessible to the collection of modelled *Physarum* agents, where “accessible” is defined as the distance of 20% of the total domain width (**e**), 10% of the total domain width (**f**), and 1% of the total domain width (**g**). The trendlines in b-c, e-g are power functions.


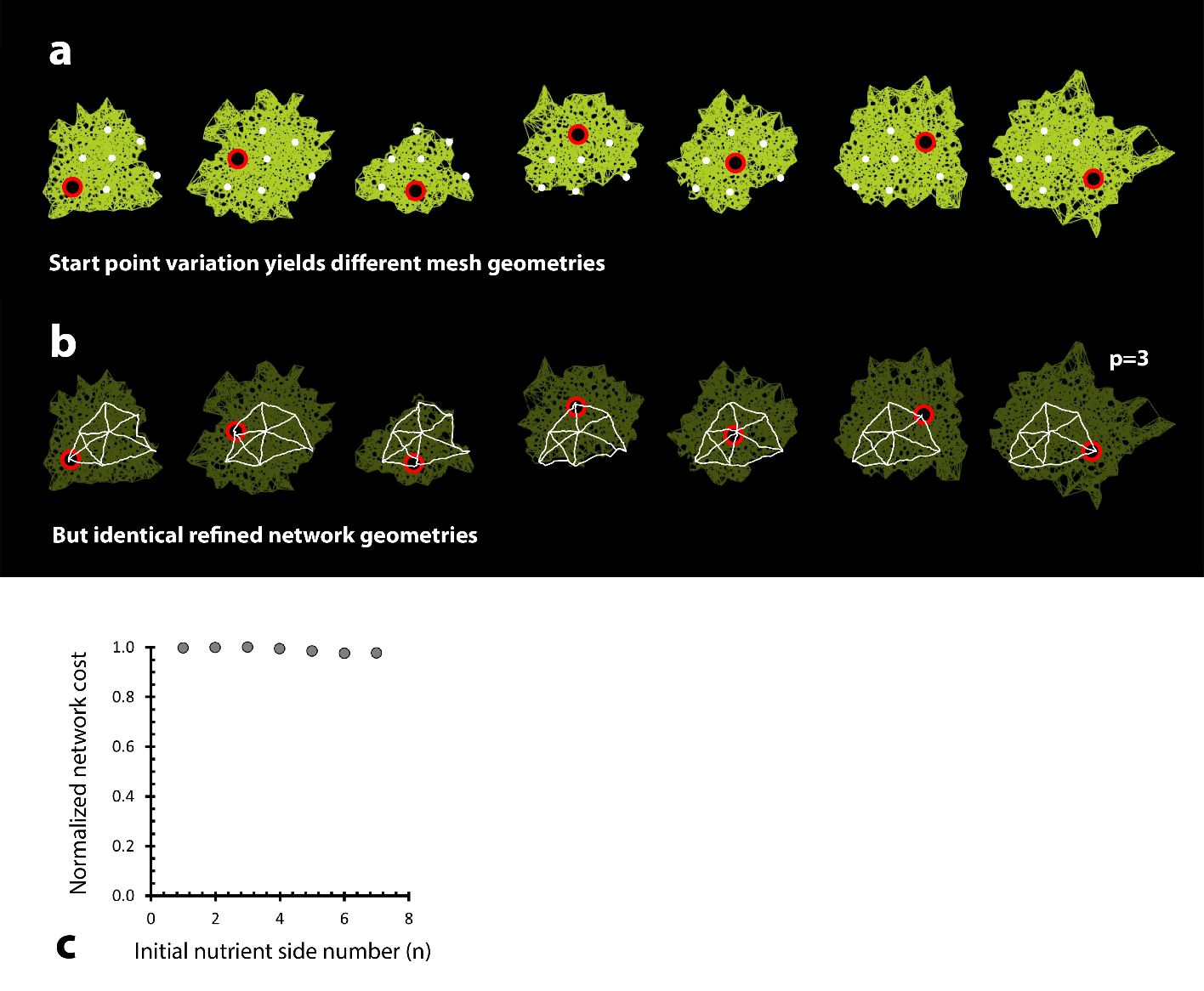


**Figure S6. Mesh morphology varies with, but refined network morphology is independent of, choice for starting node.** (**a**) Mesh geometries generated for the same set of seven attractor points, where each geometry corresponds to a simulation initialized at a unique starting point. (**b**) Final network (p=3) is nearly identical across each simulation iteration. (**c**) Final network cost varies by less than 1% between simulation iterations, and we can conclude that final network geometry is independent of choice of node for simulation initialization.


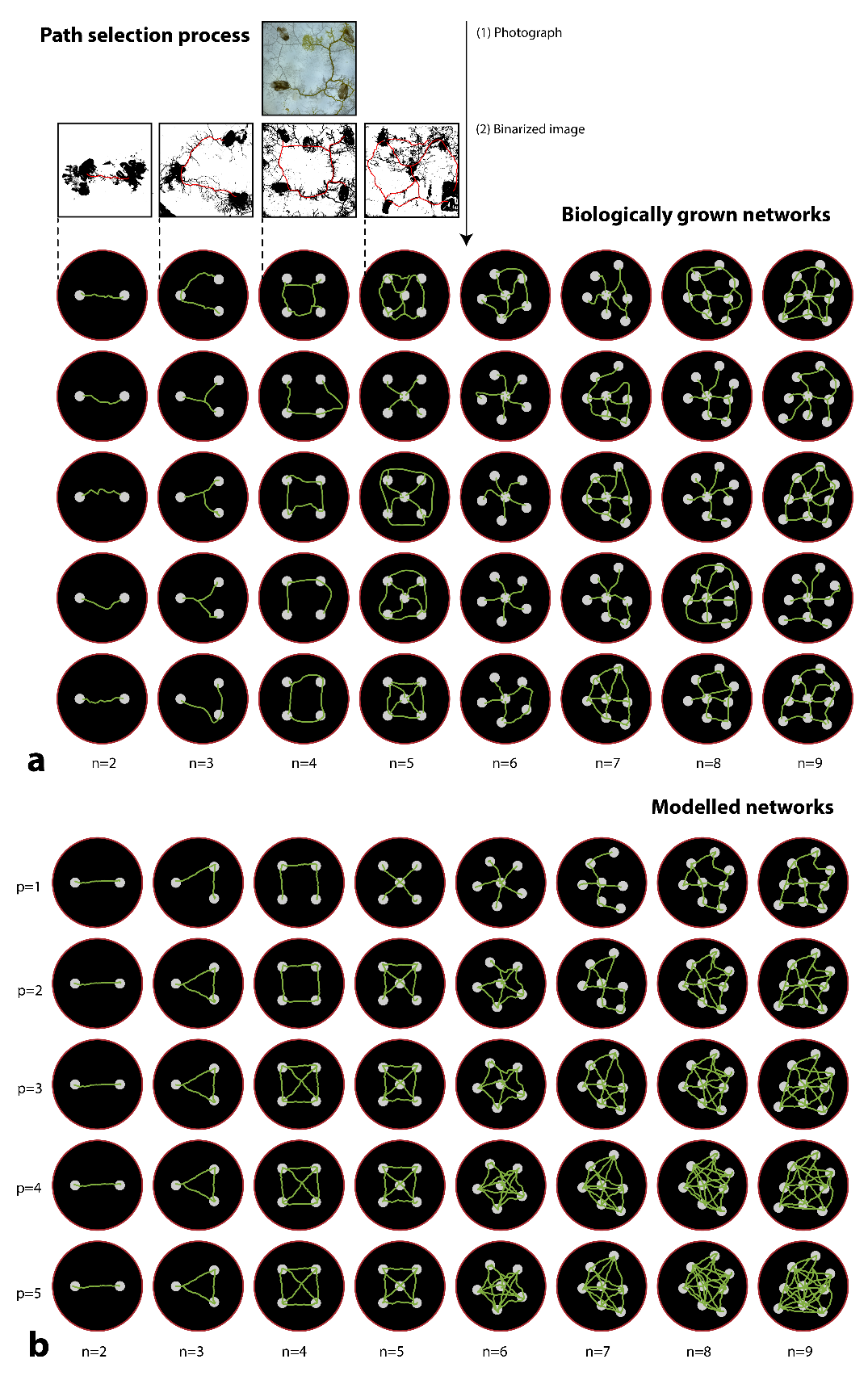


**Figure S7. Network morphologies grown (a) and simulated (b) across eight unique attractor point layouts.** Networks in (a) are grown five times for each layout, and were digitized after 5 days. Networks in (b) show one growth iteration per attractor layout with varying proximity coefficients.
